# Melioidosis Diagnostic Workshop, 2013^1^

**DOI:** 10.3201/eid2102.141045

**Published:** 2015-02

**Authors:** Alex R. Hoffmaster, David AuCoin, Prasith Baccam, Henry C. Baggett, Rob Baird, Saithip Bhengsri, David D. Blaney, Paul J. Brett, Timothy J.G. Brooks, Katherine A. Brown, Narisara Chantratita, Allen C. Cheng, David A.B. Dance, Saskia Decuypere, Dawn Defenbaugh, Jay E. Gee, Raymond Houghton, Possawat Jorakate, Ganjana Lertmemongkolchai, Direk Limmathurotsakul, Toby L. Merlin, Chiranjay Mukhopadhyay, Robert Norton, Sharon J. Peacock, Dionne B. Rolim, Andrew J. Simpson, Ivo Steinmetz, Robyn A. Stoddard, Martha M. Stokes, David Sue, Apichai Tuanyok, Toni Whistler, Vanaporn Wuthiekanun, Henry T. Walke

**Affiliations:** US Centers for Disease Control and Prevention, Atlanta, Georgia, USA (A.R. Hoffmaster, D.D. Blaney, J.E. Gee, T.L. Merlin, R.A. Stoddard, D. Sue, H.T. Walke);; University of Nevada School of Medicine, Reno, Nevada, USA (D. AuCoin);; IEM, Research Triangle Park, North Carolina, USA (P. Baccam);; Royal Darwin Hospital, Darwin, Northern Territory, Australia (R. Baird);; US Centers for Disease Control and Prevention, Nonthaburi, Thailand (H.C. Baggett, S. Bhengsri, P. Jorakate, T. Whistler);; University of South Alabama, Mobile, Alabama, USA (P.J. Brett);; Public Health England, Salisbury, UK (T.J.G. Brooks, A.J. Simpson);; University of Texas, Austin, Texas, USA (K.A. Brown);; University of Cambridge, Cambridge, UK (K.A. Brown, S.J. Peacock);; Mahidol University, Bangkok, Thailand (N. Chantratita, D. Limmathurotsakul, V. Wuthiekanun);; Alfred Health, Monash University, Melbourne, Victoria, Australia (A. C. Cheng);; Lao-Oxford-Mahosot Hospital–Wellcome Trust Research Unit, Vientiane, Laos, and University of Oxford, Oxford, UK (D.A.B. Dance);; University of Western Australia, Perth, Western Australia, Australia (S. Decuypere);; Defense Threat Reduction Agency, Fort Belvoir, Virginia, USA (D. Defenbaugh, M.M. Stokes);; InBios International, Seattle, Washington, USA (R. Houghton);; Khon Kaen University, Khon Kaen, Thailand (G. Lertmemogkolchai);; Kasturba Medical College, Manipal, India (C. Mukhopadhyay);; Townsville Hospital, Townsville, Queensland, Australia (R. Norton);; Universidade de Fortaleza, Fortaleza, Brazil (D. B. Rolim);; University of Greifswald, Greifswald, Germany (I. Steinmetz);; University of Hawaii at Manoa, Honolulu, Hawaii, USA (A. Tuanyok)

**Keywords:** melioidosis, *Burkholderia pseudomallei*, diagnosis, bacteria

## Abstract

Melioidosis is a severe disease that can be difficult to diagnose because of its diverse clinical manifestations and a lack of adequate diagnostic capabilities for suspected cases. There is broad interest in improving detection and diagnosis of this disease not only in melioidosis-endemic regions but also outside these regions because melioidosis may be underreported and poses a potential bioterrorism challenge for public health authorities. Therefore, a workshop of academic, government, and private sector personnel from around the world was convened to discuss the current state of melioidosis diagnostics, diagnostic needs, and future directions.

Melioidosis is a frequently fatal infection caused by the gram-negative bacillus *Burkholderia pseudomallei* (*1*). It is highly endemic to northeastern Thailand and northern Australia, where the causative organism is commonly found in soil and fresh water. Melioidosis also occurs in those who travel to disease-endemic regions of the world, which include tropical regions of Asia and South America, Central America, various Pacific and Indian Ocean islands, and some countries in Africa (*1*). *B. pseudomallei* can also cause latent infection; the longest documented interval between exposure and clinical melioidosis is 62 years (*2*). The crude case- fatality rate for melioidosis ranges from 14% to 40% and may be as high as 80% if effective antimicrobial drugs are not given.

Clinical diagnosis of melioidosis is difficult because the disease has no pathognomonic clinical manifestations (*1*). The current diagnostic standard is culture; however, *B. pseudomallei* can be misidentified as a culture contaminant or as another species (e.g., *Burkholderia cepacia*, *Bacillus* spp., or *Pseudomonas* spp.), especially by laboratory staff unfamiliar with this organism (*1*,*3*–*5*). In addition, *B. pseudomallei* is categorized as a Tier 1 select agent by the US government, and special precautions are recommended to reduce the possibility of exposure while conducting bacterial culture. There are currently no commercially available and reliable rapid diagnostic tests for melioidosis. Serologic tests, such as indirect hemagglutination assay (IHA), have been widely used, but these are neither sensitive nor specific.

With the goal of improving timely and accurate diagnosis of melioidosis, a workshop sponsored by the US Centers for Disease Control and Prevention was held in Bangkok, Thailand, on September 14–15, 2013, to discuss current recommendations and future research directions. International subject matter experts representing academia, government, and the private sector attended the workshop to discuss the current state of melioidosis diagnostics, diagnostic needs, and future directions. The workshop consisted of multiple sessions focused on specific diagnostic topics (e.g., culture, PCR, serology, and new methods). Each session included short presentations followed by extensive group discussions. Notes from these group discussions along with correspondence exchanged shortly after the workshop were used to clarify points and reach consensus. This article provides a workshop summary as an informative diagnostic guide for clinicians and laboratory staff.

## Optimal Diagnostic Workup for Patients with Suspected Melioidosis

Clinical manifestations of melioidosis vary widely and can include sepsis with or without a localized infection such as pneumonia or internal organ abscesses. Chronic disease (symptoms >2 months) can occur and can mimic other diseases such as tuberculosis or cancer. Therefore, melioidosis should be suspected for every patient with community-acquired sepsis, pneumonia, or abscesses, from areas where indigenous melioidosis cases have been reported. In non–meliodisis-endemic regions, such as the United States and Europe, a diagnosis of melioidosis should be considered for every patient with sepsis and a history of having traveled to melioidosis-endemic regions, especially for those with predisposing conditions such as diabetes mellitus, renal disease, or immunosuppression. Because the duration of latent infection can extend for decades (*2*), a complete travel history should be obtained. In addition, patients with no history of having traveled outside non–melioidosis-endemic regions but who have been exposed to imported animals, soil, or plants might also be at risk for *B. pseudomallei* infection (*6*,*7*), albeit rarely.

Blood culture should be performed for all patients with suspected melioidosis, and urine and throat swab samples should be obtained and cultured by using selective media, even for patients without pharyngitis or urinary symptoms (*8*–*11*). Sputum samples, swab samples from surface lesions, and aspirates of pus should be collected from patients with pneumonia, localized lesions, or abscesses and should be cultured by using selective media. Culture of rectal swab samples in selective broth may also be useful (*12*). The sensitivity of urine culture is enhanced by centrifuging and culturing the pellet (*13*). Clinicians should notify laboratories when melioidosis is suspected so laboratory scientists can perform appropriate testing and use appropriate biosafety practices to prevent laboratory exposure (*3*,*14*).

*B. pseudomallei* is able to survive for long periods in moist environments, although it survives less well at low temperatures (*15*,*16*). Although the organism may survive desiccation, viability may be compromised (*17*). Therefore, clinical samples should be transported to the laboratory at room temperature and processed as soon as possible, and swabs should preferably be placed in a suitable transport medium.

In humans, *B. pseudomallei* does not form part of the normal colonizing microbiota; growth of the organism from any site is diagnostic (*9*). Persistently positive cultures without apparent clinical disease have been described for a few patients with cystic fibrosis or bronchiectasis; however, even in these settings an attempt at eradication is worthwhile (*18*–*21*). Specimens are often culture positive even those from patients pretreated with effective antimicrobial drugs (*22*). In our collective experience, negative cultures obtained after a full diagnostic workup for patients unlikely to have melioidosis provide generally sufficient reason to cease broad-spectrum antimicrobial drugs (e.g., a carbapenem or ceftazidime) after 4–7 days. For patients with signs strongly suggestive of melioidosis, repeating all cultures on multiple occasions and searching for occult foci of infection (e.g., abscesses in liver, spleen, or urinary tract, including the prostate gland) with imaging is recommended.

## Culture and Identification

Although culture is the diagnostic standard and is 100% specific, sensitivity may be as low as 60%, depending on the method of sample collection, media used, and expertise of the microbiologist (*23*). Because many samples from patients with suspected melioidosis are collected from nonsterile sites, the use of selective media is critical. Ashdown agar is commonly used in areas where melioidosis is endemic and is cost-effective (*24*), but it is not commercially available in most countries. Alternative media that are more commonly available are *B. cepacia* selective agar and *Pseudomonas cepacia* agar (*11*,*25*). The *B. pseudomallei* load in clinical samples can vary greatly and is particularly low in blood (0.1–100 CFU/mL); the highest concentration is usually in sputum (10^2^–10^9^ CFU/mL) (*26*).

*B. pseudomallei* colonies are usually cream colored with a metallic sheen and may become dry and have a matte or wrinkled appearance after incubation for >24 hours on blood agar, although considerable variation is seen. On MacConkey agar, *B. pseudomallei* colonies are pale (lactose nonfermenters) and may exhibit a metallic sheen and become pink and umbonate or rugose after 48 hours. On triple sugar iron agar, *B. pseudomallei* may indicate either no change or slight oxidation. Nonetheless, the morphologic appearance of bacterial colonies on common culture media may also be atypical. The demonstration of typical colonies on Ashdown agar after prolonged incubation (48–96 hours) and the appearance of a pellicle in Ashdown broth add support where this medium is available (*8*,*27*). Gram-stained *B. pseudomallei* may not resemble the textbook description of having bipolar staining (“safety pin” appearance). The microscopic morphology of organisms from patients receiving antimicrobial drugs may be highly atypical, may be filamentous, or may appear similar to that of yeasts (*28*). *B. pseudomallei* is readily dismissed as a culture contaminant or misidentified as *Pseudomonas* spp. or other organisms when standard identification methods are used, including API 20NE (bioMérieux, Craponne, France) and automated bacterial identification systems (Table 1). In areas where *B. pseudomallei* is uncommonly encountered, it may be overlooked. *B. pseudomallei* colonies may resemble contaminants (e.g., *Pseudomonas stutzeri* also forms wrinkled colonies) and be discarded erroneously. Therefore, it is strongly recommended that any non–*Pseudomonas aeruginosa*, oxidase positive, gram-negative bacillus isolated from any clinical specimen should be suspected to be *B. pseudomallei* (*39*). In addition, an antibiogram may be useful for identification of oxidase-positive, gram-negative bacilli; *B. pseudomallei* is typically resistant to aminoglycosides (e.g., gentamicin), colistin, and polymyxin but susceptible to amoxicillin/clavulanic acid (*40*).

**Table 1:** Performance of commercially available systems for identification of *Burkholderia pseudomallei*

Method	Isolate source	*B. pseudomallei*, no. correct/no. tested (%)	Reference
API 20 NE*	Thailand	390/400 (98)	(*27*)
API 20 NE	Singapore	40/50 (80)	(*29*)
API 20 NE	Australia	101/103 (98)	(*30*)
API 20 NE	Australia	26/71 (37)	(*31*)
API 20 NE	United States (imported)	35/58 (60)	(*32*)
API 20 NE	Thailand/various	792/800 (99)	(*33*)
Phoenix†	Singapore	13/47 (28)	(*34*)
Phoenix	Malaysia/Thailand	0/1 (0)	(*35*)
VITEK 2*	Australia	19/103 (19)	(*30*)
VITEK 2	Australia	83/103 (81)	(*36*)
VITEK 2	Malaysia	0/1 (0)	(*37*)
VITEK 2	Australia	146/149 (98)	(*38*)
VITEK 2	Sabah, Malaysia	22/25 (88)	(*38*)
VITEK 2	Sarawak, Malaysia	23/43 (53)	(*38*)

*bioMérieux, Craponne, France. †Becton, Dickinson, and Company, Franklin Lakes, NJ, USA.

Latex agglutination is particularly useful as a rapid diagnostic test for the identification of *B. pseudomallei* isolates grown on solid agar or liquid culture or directly on blood culture fluid. The latex agglutination reagent developed in Thailand, based on a monoclonal antibody specific to a 200-kDa exopolysaccharide, has a sensitivity of 95.1% and specificity of 99.7% on blood culture fluid (*41*). Several other latex agglutination assays that use monoclonal or polyclonal antibodies developed in house have been described; however, comparative performance of these assays in routine clinical practice has not been undertaken to date (*39*,*42*,*43*). The use of a validated, specific latex agglutination reagent is sufficient for identifying isolates suspected to be *B. pseudomallei* on the basis of the microbiological characteristics described above. Any atypical isolates that are potentially *B. pseudomallei*, and the first such isolates from any geographic region, should ideally undergo further confirmatory testing. Latex agglutination in particular fulfills many of the characteristics of a useful test; it is rapid (<5 minutes), simple to learn, and inexpensive; results are reproducible and accurate. It can enable technicians in local microbiology facilities in developing countries to identify *B. pseudomallei* effectively. We support initiatives to improve the availability of such a test worldwide, which ideally could be used to screen all suspect *B. pseudomallei* originating from clinical specimens.

In general, commercially available identification systems (e.g., API 20NE, Phoenix [Becton, Dickinson and Company, Franklin Lakes, NJ, USA], and VITEK [bioMérieux]) perform adequately (Table 1). Fresh cultures should be used for biochemical testing, and it is important to note the apparent regional variation in performance of some identification kits (*30*). The API 20NE correctly identified 98%–99% of *B. pseudomallei* isolates in Thailand but identification was highly variable (37%–98%) in Australia, where *B. pseudomallei* was commonly misidentified as *B. cepacia* or *Chromobacterium violaceum* (*27*,*30*,*31*,*33*,*44*). In addition, isolates from Malaysia are more commonly misidentified because they are poorly represented in biochemical profile databases and may be susceptible to gentamicin, issues that could be important when considering strains from other locations (*38*). Misidentification may lie with the interpretation of assimilation tests, which can be difficult to read when using API 20NE (*33*). Previous reports showed that VITEK 1 correctly identified 99% of *B. pseudomallei* isolates. The fluorometric-based ID–gram-negative bacillus card of the VITEK 2 correctly identified only 19% of *B. pseudomallei* in 2002, but a newer colorimetric-based GN (gram-negative) card identified 63%–81% of *B. pseudomallei* correctly, depending on the culture media used (*30*,*36*). Automated systems accuracy relies on the size of the strain database used for identification.

Where reference laboratories are available, definitive species identification is possible by PCR with use of a variety of published systems such as TTS1, BurkDiff, and others (*45*–*47*). The Laboratory Response Network *Burkholderia* spp. real-time PCR assay is also available in laboratories participating in the Network (*48*). Laboratories with sequencing capabilities may also consider using 16S rRNA gene sequencing (*49*).

Disk-diffusion susceptibility testing is routinely used in melioidosis-endemic areas, although as yet no interpretative criteria have been published by the Clinical and Laboratory Standards Institute, which recommends measurement of MICs for *B. pseudomallei* (*50*). A specific issue arises when performing antimicrobial drug–susceptibility testing for co-trimoxazole, a first-line antimicrobial drug used in the eradication phase of melioidosis treatment. Testing should use a MIC-based method because the disk-diffusion method overestimates resistance (*51*–*53*). Graduated antibiotic strips (Etests) may be used but are sometimes difficult to read because of the “double zone,” a phenomenon that occurs when combination antimicrobial drug formulations are tested.

## Rapid Detection of *B. pseudomallei* in Clinical Specimens

Several assays that can be used for direct detection of *B. pseudomallei* in clinical specimens have been developed and include an immunofluorescence assay (IFA), PCRs, and a lateral flow immunoassay (LFI). In addition, these tests can be used for the identification of *B. pseudomallei* isolates grown on solid agar or in liquid culture.

The IFA is rapid, simple, and reliable and uses a monoclonal antibody against capsule polysaccharide (CPS) to detect *B. pseudomallei* directly in clinical specimens or from blood culture bottles (*28*,*54*). It is particularly useful for specimens in which bacterial density is at least 10^3^ CFU/mL (e.g., in pus, sputum, and urine) (*28*). Although culture results may take 1–7 days, IFA takes only 15 minutes. However, IFA requires a UV microscope and experienced technicians, and the diagnostic sensitivity of IFA (range 45%–66%) is lower than that of culture (*28*,*55*). Although IFA is not commercially available, it has a long, positive track record of use in some specialized laboratories for providing rapid diagnosis in melioidosis-endemic regions.

Nucleic acid detection methods could shorten the time to diagnosis. Several PCRs have been developed and evaluated, including conventional and real-time PCRs; the latter PCR is more rapid and sensitive. Some assays detect *B. pseudomallei* exclusively, whereas others are designed in multiplex formats to identify *B. pseudomallei* and differentiate it from close relatives such as *Burkholderia mallei* or *Burkholderia thailandensis* (*45*). To date, these assays have been useful for identifying isolates (*47*,*56*), but their performance in testing DNA extracted directly from specimens has been variable, and they are not routinely used in melioidosis-endemic regions (*31*,*47*,*56*–*58*). Some specimens (e.g., sputum) are more likely to yield a positive result than are others (e.g., blood), probably because of differing bacterial concentrations in these specimens (*57*–*59*).

An LFI has been developed that uses a monoclonal antibody specific to CPS similar to that used in the latex agglutination test (*60*). The assay has been shown to work with various types of clinical specimens routinely collected from patients with suspected melioidosis and to identify the organism isolated from solid and liquid media. Sensitivity and specificity of the LFI have been evaluated on 77 diverse *B. pseudomallei* isolates and 36 near-neighbor species and were 98.7% and 97.2%, respectively. A single atypical isolate that had a mutation reported to affect CPS expression produced a false-negative result, and a single *B. thailandensis* isolate that had the CPS biosynthetic operon and expresses capsule produced a false-positive result (*20*,*60*,*61*). Most *B. thailandensis* strains do not have this operon; in addition, this species is not typically associated with infections and is thus unlikely to cause false-positive results in the clinical laboratory (*61*). This test has the potential for use as a rapid diagnostic test for *B. pseudomallei* identification worldwide.

## Serologic Tests

The IHA is the main serologic assay used worldwide, although it lacks standardization. The diagnostic sensitivity of the IHA at admission is only 56%, and the variable prevalence of background seropositivity in areas where melioidosis is endemic reduces its specificity (*62*–*64*). As a result, the IHA has no role in the diagnosis of melioidosis in disease-endemic regions, and its use should be discouraged. The IHA may be of value during the evaluation of febrile illness in travelers who have not lived in but have traveled to a melioidosis-endemic region. A negative result does not rule out melioidosis, but a positive result implies exposure to *B. pseudomallei* (*65*). The IHA is also useful in non–melioidosis-endemic areas for potentially exposed laboratory workers or military personnel (*3*,*66*). Although a 4-fold rise in IHA titer has been used as evidence of melioidosis infection, this finding is not sufficiently sensitive or specific enough to guide treatment decisions in melioidosis-endemic areas. Similarly, although titers might wane after treatment, a persistently high IHA titer does not necessarily indicate treatment failure or latent infection (*62*).

Other serologic assays, including in-house tests using ELISA, have been developed (*67*). However, development and evaluation of serologic tests have been hampered by the low sensitivity of the diagnostic standard (i.e., culture) (*23*,*68*). ELISA is a much less labor-intensive assay for some applications. Work with latent class statistical models has raised the possibility that culture is an imperfect diagnostic standard (*23*). This finding has prompted reevaluation of older serologic assays (*68*) and has implications for the evaluation of new diagnostic tests (*69*). Tools for analyzing diagnostic test data where there are no diagnostic standards have been made available online (http://mice.tropmedres.ac) (*70*). In addition, new serologic assays that use polysaccharides purified from *B. pseudomallei*, such as O-antigen polysaccharide and CPS, are being developed. These tests have the potential to be the next generation of serologic assays and will enable greater standardization. Multiplex assays are also being developed to detect *B. pseudomallei* antigens and antibody in combination with tests for other pathogens.

## Future Diagnostic Tests

Matrix-assisted laser desorption/ionization time-of-flight mass spectrometry (MALDI-TOF) is increasingly being used as a rapid method for isolate identification. This method requires comparison of mass spectroscopy profiles against a database of isolates belonging to known species. There are 2 types of database: a closed database for which the fidelity of isolates is verified by the manufacturer and an open database to which isolates are added locally. The performance of MALDI-TOF is hampered by the sparse number of isolate profiles in current closed databases (*71*). Efforts to add *B. pseudomallei* isolates to local open databases are under way in some melioidosis-endemic areas, but their provenance must be clear. Addition of these isolates to closed proprietary databases would make them more useful outside melioidosis-endemic areas. Although there is a proliferation of new species within the genus *Burkholderia* for which no profiles exist on MALDI-TOF databases, the clinical significance of these species is borderline because few are associated with clinical disease.

MALDI-TOF methods are also being used to detect unique metabolite signatures present in patients with melioidosis. Preliminary work indicates that the metabolome of patients with melioidosis can be differentiated from that of patients with sepsis from other causes. The identification of such metabolites could lead to the development of rapid assays for their specific detection.

Also being developed are rapid antimicrobial drug–susceptibility testing methods that use quantitative PCR to rapidly evaluate susceptibility by comparing the growth of bacteria exposed to varying concentrations of antimicrobial drugs with that of unexposed bacteria. These methods are being developed as part of bioterrorism preparedness initiatives in the United States to ensure rapid and appropriate responses. According to preliminary work, the results are available up to 12 hours sooner and seem to correlate with conventional broth microdilution results for many, but not all, clinically relevant antimicrobial drugs. This approach has been used successfully for *Bacillus anthracis* (*72*).

## Misconceptions and Pitfalls when Diagnosing Melioidosis

In many regions of the world, the lack of microbiology laboratories hampers the diagnosis of bacterial infections in general, including melioidosis. Nonetheless, where microbiology facilities exist, identification of patients with melioidosis can still be problematic because of the lack of awareness among clinicians and laboratory staff, including lack of awareness of low-level endemicity where indigenous cases have been described (e.g., in India and Brazil) and failure to elicit or communicate a history of travel from patients returning from melioidosis-endemic areas. Table 2 describes common misconceptions and pitfalls that can occur when diagnosing melioidosis; the Figure illustrates when to suspect melioidosis, what specimens to take, and what types of tests are available.

**Table 2:** Common misconceptions and pitfalls in the identification of *Burkholderia pseudomallei* and diagnosis of melioidosis

Misconception or pitfall	Comments
Melioidosis is endemic only to some parts of Asia and northern Australia.	Melioidosis is reported in many regions of the world, including regions of Central and South America, various Pacific and Indian Ocean islands, and some countries in Africa.
Melioidosis is not endemic to the area because *B. pseudomallei* has never been reported from the microbiological facilities.	*B. pseudomallei* can be misidentified as another *Burkholderia* species, *Pseudomonas* spp., or other organisms, especially by laboratory staff unfamiliar with *B. pseudomallei.*
Melioidosis is only an acute, septic illness.	10%–15% of patients have chronic disease that may mimic other conditions, including tuberculosis.
Lifetime travel history to non–melioidisos-endemic areas is not taken.	Melioidosis may appear many years after exposure.
Do not provide treatment for melioidosis unless any diagnostic test is positive.	Melioidosis is often fatal, and treatment effective against *B. pseudomallei* should be provided immediately if melioidosis is suspected.
Throat swab and urine specimens should be collected only from patients with symptoms of pharyngitis or urinary tract infection.	Swabs of throat (anterior fauces) or urine may be positive in patients without focal symptoms.
Culture is a sensitive method for diagnosing melioidosis.	As with most infections, the sensitivity of culture depends on the quality of the specimen, and deep, occult sites of infection are also possible.
Indirect hemagglutination assay is a reliable diagnostic test.	Sensitivity and specificity of indirect hemagglutination assay is poor.
*B. pseudomallei* can be a colonizing organism.	Although chronic infection after treatment has been described, isolation of *B. pseudomallei* from any body site should be regarded as indicative of disease.
Selective media for *B. pseudomallei* are not necessary.	Sensitivity of culture is lower and the diagnosis would be missed for many patients if selective media are not used for specimens from nonsterile sites.
The “safety pin” appearance is a reliable characteristic of gram-stained *B. pseudomallei.*	*B. pseudomallei* usually stains unevenly but is not always bipolar, whereas other organisms such as *Eshcherichia coli* or *Klebsiella* spp. may appear bipolar with gram stain.
Automated microbiology systems can reliably detect *B. pseudomallei.*	Although these systems are generally reliable, misidentification is not uncommon, particularly in regions where few strains are included in phenotypic databases.

**Figure:**
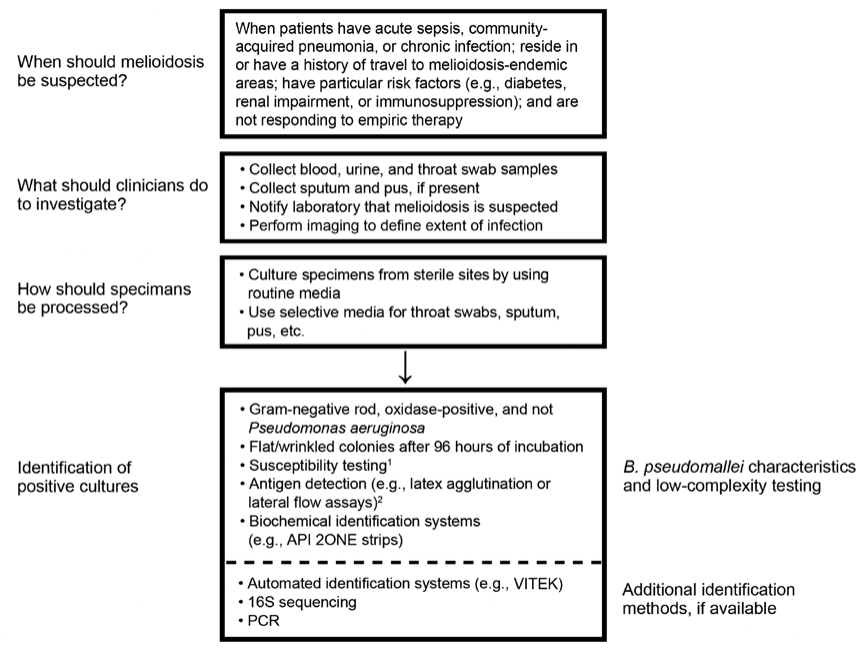
Diagnostic guidelines for clinicians and microbiologists in developed countries and resource-limited settings. 1) The antimicrobial drug–susceptibility pattern can be useful for distinguishing *Burkholderia pseudomallei* (usually resistant to aminoglycosides and colistin or polymyxin but susceptible to amoxicillin/clavulanic acid) from other pathogenic species. However, isolates can occasionally be susceptible to aminoglycosides; susceptibility may vary by region (*40*). If disk diffusion is used, zone diameter interpretation may need to be modified from break points recommended for *Enterobacteriaceae* by the Clinical and Laboratory Standards Institute (*73*). 2) Not currently commercially available. API 20NE, bioMérieux, Craponne, France; VITEK, Becton, Dickinson, and Company, Franklin Lakes, NJ, USA.

## Challenges in Various Settings

In melioidosis-endemic areas, if melioidosis is suspected, empiric treatment with antimicrobial agents effective against *B. pseudomallei* should be initiated immediately, before diagnostic results are available, in an effort to reduce the number of deaths. Diagnostic tests used in melioidosis-endemic areas should be able to confirm melioidosis with high accuracy, with high positive and negative predictive values. High positive and negative predictive values are essential if the test result is being used to determine whether melioidosis-specific antimicrobial agents (rather than broad-spectrum empirically used antimicrobial agents to cover melioidosis-specific and other pathogenic organisms) are appropriate and whether the patients need to be treated with prolonged oral therapy to prevent melioidosis relapse. A rapid test that could be used at the point of care would be most useful in melioidosis-endemic areas. The ideal rapid test should use inexpensive commonly available equipment, supplies, and reagents. It should require minimal training, be robust in a variety of laboratory conditions (temperature, humidity), and have a long shelf life. It should be accurate and reliable even when performed on direct specimens, to minimize the hazard of working with pure culture.

In areas where melioidosis is less common or in non–melioidosis-endemic areas, empiric antimicrobial therapy for acute sepsis may not include drugs active against *B. pseudomallei*. In addition, the positive predictive values of rapid tests are probably much lower because of the low-prevalence setting. Therefore, diagnostic tests developed for these regions should focus on methods that detect pathogens more broadly and include *B. pseudomallei*, such as 16S sequencing or multiplexed real-time PCR assays. A combination of antigen and antibody detection to provide high specificity and sensitivity might be a possible solution for this setting. Educating technicians and clinicians about diagnosis of melioidosis is also necessary. Reporting of cases that occur in areas where melioidosis is less common or in non–melioidosis-endemic areas might help familiarize technicians and clinicians with this pathogen and alert public health officials to potential outbreaks.

## Conclusions

The timely and accurate diagnosis of melioidosis is needed to ensure that effective antimicrobial therapy is initiated or continued appropriately. Distinct diagnostic obstacles exist in settings where melioidosis is or is not endemic and in environments with low or high levels of resources. Common misconceptions and pitfalls relating to diagnostic microbiology can also hinder early detection. Efforts to culture *B. pseudomallei* from persons suspected to have melioidosis are paramount and should include culturing of all available specimens by using selective media such as Ashdown agar or *B. cepacia* agar. The need to make latex agglutination testing available for rapid identification of isolates, particularly in low-resource melioidosis-endemic areas, received widespread support. Simple point-of-care tests such as the LFI may become available in the near future and would enable rapid identification of isolates and direct detection in clinical specimens. This capacity will greatly aid rapid diagnosis in developed countries and in low-resource settings.
